# A diagnostic pitfall in iron-refractory microcytic hypochromic anemia with acquired ring sideroblasts initially treated as iron deficiency anemia—a case report

**DOI:** 10.3389/fmed.2026.1838995

**Published:** 2026-06-08

**Authors:** Xiaoqi Liu, Juning Ma, Anning Song, Sheng Yang, Mingxia Li, Jing Yang

**Affiliations:** 1 College of First Clinical Medicine, Shandong University of Traditional Chinese Medicine, Jinan, China; 2 Weifang Traditional Chinese Hospital, Weifang, China

**Keywords:** diagnostic reassessment, microcytic hypochromic anemia, pyridoxine, ring sideroblasts, sideroblastic anemia

## Abstract

Microcytic hypochromic anemia is commonly presumed to represent iron deficiency anemia (IDA) at initial presentation. However, failure to respond to iron supplementation and iron indices inconsistent with IDA should prompt early diagnostic reassessment. We report a 67-year-old woman with severe microcytic hypochromic anemia after ineffective iron therapy. Iron studies indicated iron loading with impaired iron utilization rather than iron deficiency. Bone marrow Perls staining revealed ring sideroblasts in 14% of erythroblasts. Further stratified evaluation supported an acquired ring sideroblast phenotype, but the etiology could not be conclusively established. The patient received multifactorial supportive management, including discontinuation of unnecessary iron supplementation, early transfusion support, erythropoietin during hospitalization, pyridoxine supplementation, infection treatment, and optimization of comorbidities. Hemoglobin gradually improved and remained stable during follow-up without further transfusion. This case underscores the importance of early reassessment in older patients with iron-refractory microcytic anemia and discordant iron indices. Bone marrow Perls staining is critical for identifying ring sideroblasts. Assessment including SF3B1 testing may aid etiologic stratification, but SF3B1 negativity alone is insufficient to exclude clonal myeloid disease.

## 1 Introduction

Microcytic hypochromic anemia is a common hematologic presentation, but the presence of microcytosis and hypochromia alone is insufficient for etiologic diagnosis. In addition to iron deficiency anemia (IDA), sideroblastic anemia (SA), anemia of chronic disease, and other conditions may also present with a microcytic pattern. SA is characterized by the presence of ring sideroblasts (RS) on bone marrow iron staining, reflecting impaired iron utilization during erythropoiesis despite preserved or increased iron stores (1, 2). SA is a heterogeneous entity that includes both hereditary and acquired forms. Acquired SA comprises both clonal and non-clonal causes, including potentially reversible causes such as drugs, toxins, and nutritional deficiencies. (3–5).

Accurate diagnostic reconstruction is particularly important in older patients with multimorbidity, in whom inflammation may distort iron indices, especially ferritin and transferrin, making ferritin-based reasoning alone unreliable (6). Current guidance therefore favors panel-based interpretation of iron status, including transferrin saturation and iron-binding capacity indices, with careful interpretation under inflammatory conditions (7, 8). Pyridoxine responsiveness is also mechanistically plausible in selected sideroblastic phenotypes, as pyridoxal 5′-phosphate, the active form of vitamin B6, is an essential cofactor for ALAS2, the first and rate-limiting enzyme in heme biosynthesis (9, 10). Nevertheless, hematologic improvement during hospitalization may be confounded by transfusion support, erythropoietin use, and optimization of comorbid conditions, such that treatment attribution should rely on longitudinal follow-up rather than short-term hemoglobin changes alone.

**Figure 1:**
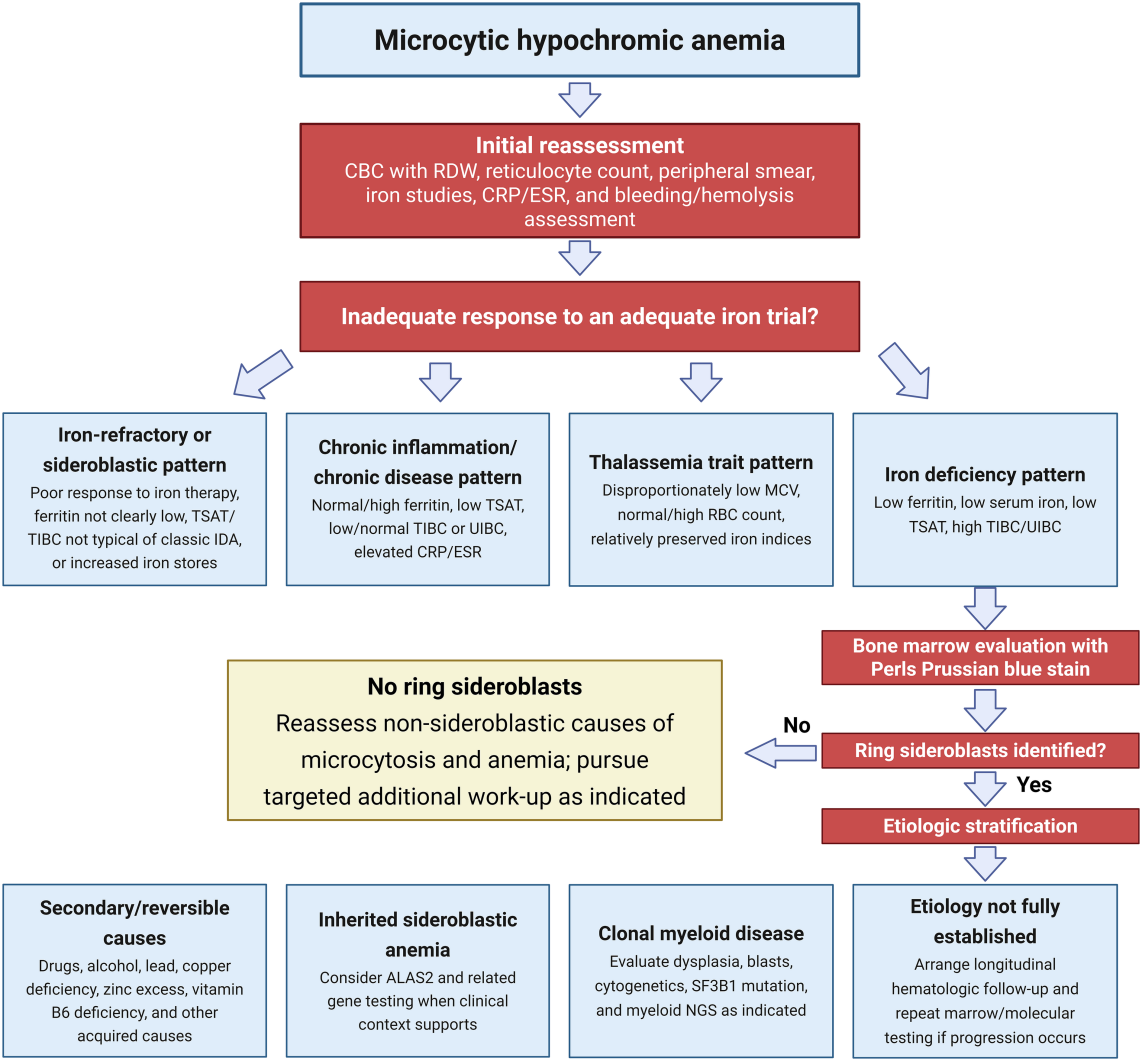
Reassessment pathway for microcytic hypochromic anemia with inadequate response to iron therapy. The algorithm emphasizes reassessment of iron indices, inflammatory status, thalassemia-related features, bleeding/hemolysis, and other non-sideroblastic causes before bone marrow evaluation. In patients with discordant iron profiles or poor hematologic response to an adequate iron trial, bone marrow Perls Prussian blue staining may help identify ring sideroblasts. When ring sideroblasts are present, etiologic stratification should consider secondary/reversible causes, inherited sideroblastic anemia, clonal myeloid disease, and cases in which the etiology remains incompletely established. Ferritin should be interpreted cautiously in the presence of inflammation, and pyridoxine responsiveness alone should not be regarded as definitive proof of etiology.

We report an older woman with multisystem comorbidities whose severe microcytic hypochromic anemia was initially treated as IDA without benefit. Subsequent bone marrow iron staining and etiologic stratification redirected the diagnosis toward an acquired sideroblastic phenotype. This case highlights the importance of early diagnostic escalation when phenotype, iron studies, and treatment response are discordant, and provides the basis for a simplified diagnostic pathway for microcytic anemia in older patients with multimorbidity (Figure 1).

## 2 Case presentation

### 2.1 Patient information and clinical presentation

A 67-year-old woman was admitted with progressively worsening anemia-related symptoms over 1 month. Her anemia had been incidentally detected during a prior hospitalization for diabetic ketoacidosis 1 month earlier. At that time, the family declined bone marrow examination, and she was presumptively treated for iron deficiency anemia (IDA) on the basis of a microcytic hypochromic phenotype. After approximately 1 month of oral iron supplementation, no clinical improvement was observed.

Her medical history included type 2 diabetes mellitus for 10 years with poor glycemic control and hypertension for more than 20 years, with irregular antihypertensive treatment. She denied hematemesis, melena, toxic exposure, smoking, and alcohol consumption. There was no family history of hereditary anemia or hematologic disease. On admission, she reported marked fatigue. Physical examination revealed severe pallor without superficial lymphadenopathy, hepatosplenomegaly, or sternal tenderness. Vital signs were stable.

A detailed medication, exposure, nutritional, and family history review was performed to assess potentially reversible or secondary causes of the acquired ring sideroblast phenotype (Supplementary Table S1). Before admission, the patient had received insulin aspart, insulin glargine, and linagliptin for type 2 diabetes mellitus, as well as amlodipine besylate for hypertension. She had also received empiric oral polysaccharide-iron complex for approximately 1 month before admission. No exposure to classic drugs associated with acquired sideroblastic anemia, including isoniazid, linezolid, chloramphenicol, antidepressants, or other psychotropic medications, was identified. The patient also denied alcohol, lead, or other toxic exposures, and no use of zinc-containing supplements or denture adhesives was reported. Vitamin B12 and folate levels were within the laboratory reference ranges, and there was no family history of hereditary anemia or hematologic disease.

**Figure 2:**
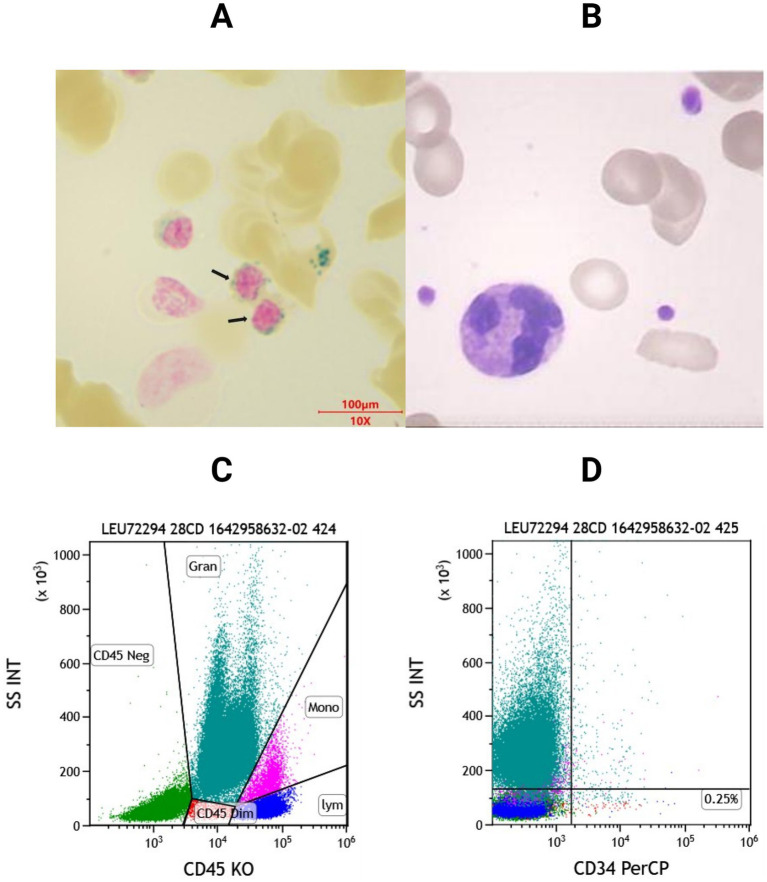
Bone marrow, peripheral blood smear, and flow cytometric findings. **(A)** Bone marrow Perls Prussian blue staining showed markedly increased iron stores and ring sideroblasts (arrows). **(B)** Peripheral blood smear showed microcytic hypochromic erythrocytes with anisocytosis (Wright–Giemsa stain, ×1,000). **(C)** Bone marrow flow cytometry showed the CD45 versus side-scatter overview gating pattern. **(D)** Bone marrow flow cytometry showed CD34-positive cells accounting for 0.25% of nucleated cells, without major abnormal immunophenotypic findings.

Initial laboratory evaluation revealed severe microcytic hypochromic anemia (hemoglobin, 46 g/L) with leukopenia, while the platelet count remained within the normal range. Peripheral blood smear demonstrated microcytic hypochromic erythrocytes with anisocytosis (Figure 2B). Iron studies were not compatible with typical iron deficiency anemia. Both fecal occult blood testing and the direct antiglobulin test (DAT) were negative. Renal function, bilirubin fractions, reticulocyte percentage, absolute reticulocyte count, and lactate dehydrogenase (LDH) did not support renal anemia or active hemolysis (Table 1).

**Table 1:** Key laboratory findings at admission relevant to diagnostic reassessment.

Diagnostic domain	Parameter	Result	Unit	Reference range
Hematologic profile	Hemoglobin	46	g/L	115–150
	Red blood cell count	1.8	×10^12^/L	3.80–5.10
	Mean corpuscular volume	79.7	fL	82–100
	Mean corpuscular hemoglobin	25.3	pg	27–34
	White blood cell count	2.28	×10^9^/L	3.5–9.5
	Platelet count	180	×10^9^/L	125–350
	Reticulocyte percentage*	0.12	%	0.5–1.5
	Absolute reticulocyte count*	0.0029	×10^12^/L	0.02–0.2
Iron status	Serum iron	37.6	μmol/L	7.8–32.2
	Ferritin	217.8	μg/L	13–150
	Transferrin saturation	79.8	%	20–55
	Unsaturated iron-binding capacity	9.5	μmol/L	31–51
Inflammatory markers	Erythrocyte sedimentation rate	119	mm/h	0–20
	High-sensitivity C-reactive protein	9.98	mg/L	—
Tests relevant to differential diagnosis	Erythropoietin	45.73	mIU/mL	2.59–18.50
	Vitamin B12	259.977	pg/mL	200–900
	Folate	12.438	nmol/L	6.8–36.3
	Creatinine	42	μmol/L	30–80
	Lactate dehydrogenase	139	U/L	109–245
	Total bilirubin	13	μmol/L	5.1–24.1
	Direct bilirubin	4.6	μmol/L	0–6.8
	Indirect bilirubin	8.4	μmol/L	0.0–17.0
	Direct antiglobulin test	Negative	—	Negative
	Fecal occult blood test	Negative	—	Negative

1. Values are from admission unless otherwise indicated. Reference ranges are laboratory-specific.

2. “Negative” and “positive” indicate qualitative results as reported by the hospital laboratory.

3. Iron indices are reported in SI units; transferrin saturation is expressed as a percentage.

^*^Reticulocyte percentage and absolute reticulocyte count were measured on hospital day 3.

### 2.2 Bone marrow evaluation and diagnostic workup

Given the lack of response to iron therapy and the discordance between the iron profile and a typical iron-deficient phenotype, bone marrow morphologic examination and iron staining were performed. The bone marrow aspirate showed markedly active marrow with a G: E ratio of 0.75:1, indicating erythroid-predominant hyperplasia. The differential count was based on 200 nucleated marrow cells. No blasts were recorded in the 200-cell bone marrow differential count, and no increase in blasts was reported. Granulocytic cells at different maturation stages showed no obvious morphologic abnormalities. The erythroid lineage was markedly active and was mainly composed of intermediate and late erythroblasts, with a small number of erythroblasts showing nuclear irregularities. A total of 58 megakaryocytes were counted, and no obvious megakaryocytic morphologic abnormality was reported. Perls Prussian blue staining demonstrated markedly increased extracellular iron stores (+++) and ring sideroblasts accounting for 14% of erythroid precursors (Figure 2A). Detailed bone marrow smear findings are summarized in Supplementary Table S5.

To further clarify the etiology and assess the possibility of MDS-related disease, SF3B1 testing and bone marrow flow cytometric immunophenotyping were performed. No SF3B1 mutation was detected. Flow cytometry showed the overview gating pattern on CD45 versus side scatter and demonstrated that CD34-positive cells accounted for 0.25% of nucleated cells, with no immunophenotypic evidence of acute leukemia, lymphoma, plasma cell neoplasm, or high-risk MDS-associated abnormalities (Figures 2C,D). Taken together, the available findings supported a sideroblastic process, characterized by increased marrow iron stores and 14% ring sideroblasts. However, because cytogenetic analysis, expanded myeloid NGS, serum copper/zinc assessment, baseline PLP measurement, ALAS2 germline testing, and repeat marrow evaluation were not available due to insurance coverage, economic burden, and real-world clinical feasibility, the precise etiology of the ring sideroblasts could not be definitively established. Nevertheless, SF3B1 mutation testing was negative, and flow cytometry showed no immunophenotypic evidence of acute leukemia, lymphoma, plasma cell neoplasm, or high-risk MDS-associated abnormalities. In addition, medication and exposure review did not identify classic offending drugs, alcohol, lead, zinc-containing supplements, or denture adhesive exposure. Therefore, the case was interpreted as an acquired ring sideroblast phenotype of undetermined etiology rather than definitive MDS or proven non-clonal sideroblastic anemia. Longitudinal follow-up was planned, with repeat bone marrow evaluation, cytogenetic analysis, and expanded molecular testing to be considered if cytopenias recurred, persisted, or worsened.

### 2.3 Treatment and follow-up

Iron supplementation was discontinued. Given the presence of severe symptomatic anemia (hemoglobin, 46 g/L), the patient received transfusion support with 5 units of leukoreduced packed red blood cells, including 2 units on November 16, 2023, and 3 units on November 17, 2023. A therapeutic trial of intravenous pyridoxine (vitamin B6) was initiated during hospitalization at a dose of 300 mg/day, followed by oral pyridoxine at 100 mg/day after discharge. Erythropoietin was administered as supportive treatment during hospitalization. Concomitant management included intensive glycemic control, antihypertensive therapy, correction of electrolyte disturbances, and treatment of a mild urinary tract infection.

**Figure 3:**
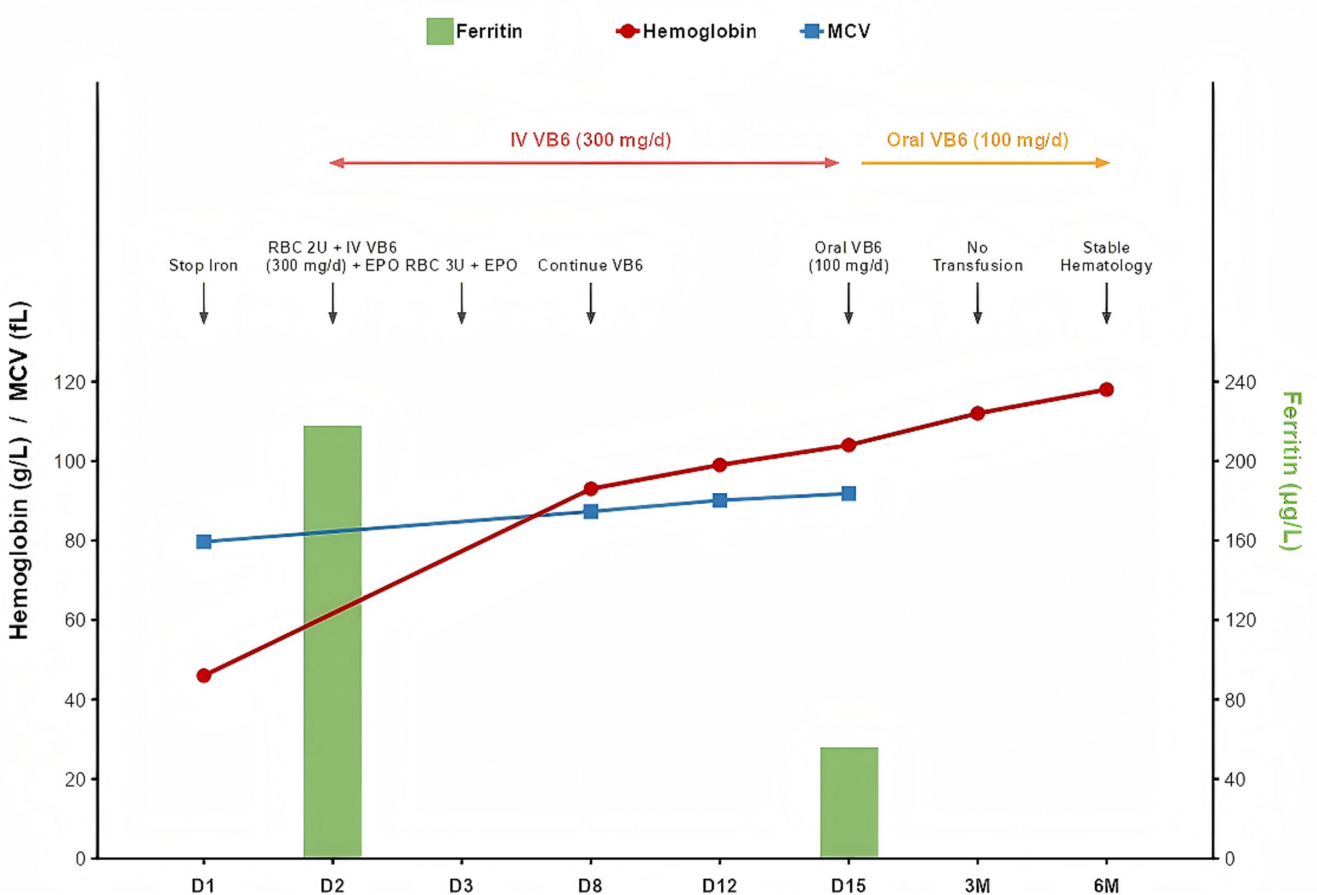
Longitudinal changes in hematologic indices during hospitalization and follow-up after multifactorial supportive management.

Hemoglobin increased to 93 g/L on hospital day 7 (November 21, 2023). Because the patient received 5 units of leukoreduced packed red blood cells during the first 3 hospital days, the early increase in hemoglobin from 46 g/L to 93 g/L was largely compatible with the expected transfusion-related increment. However, after discharge, no additional transfusions were administered, and hemoglobin remained stable at 112 g/L at approximately 3 months and 118 g/L at approximately 6 months. Therefore, the early inpatient hemoglobin rise and the sustained follow-up stability were interpreted separately: the early rise was mainly transfusion-associated, whereas the later stability occurred after multifactorial supportive management, including pyridoxine supplementation and comorbidity management (See Figure 3).

The timeline indicates the main interventions, including discontinuation of iron supplementation, red blood cell transfusion, erythropoietin administration during hospitalization (9,000 IU on D2 and 6,000 IU on D3), intravenous pyridoxine at 300 mg/day during hospitalization, oral pyridoxine maintenance at 100 mg/day after discharge, and the absence of further transfusion during follow-up. The patient received 5 units of packed red blood cells during the first 3 hospital days, including 2 units on hospital day 2 and 3 units on hospital day 3. Hemoglobin increased from 46 g/L at admission to 93 g/L on hospital day 7, representing an observed increase of 47 g/L, which was broadly compatible with the expected transfusion-related increment. The later course showed transfusion-free hematologic stability, with hemoglobin levels of 112 g/L at approximately 3 months and 118 g/L at approximately 6 months. The red line represents hemoglobin, the blue line represents MCV, and the green bars represent ferritin.

## 3 Discussion

### 3.1 Diagnostic workflow and case interpretation

This case highlights a common diagnostic pitfall in clinical practice, namely that microcytic hypochromic anemia may be prematurely attributed to iron deficiency anemia (IDA) when assessed on the basis of phenotype alone. In clinical practice, two findings should prompt reconsideration of the initial diagnosis and escalation of the diagnostic work-up: lack of response to an adequate trial of iron therapy and an iron profile that is discordant with classic IDA. In particular, elevated transferrin saturation together with low unsaturated iron-binding capacity and a ferritin level that is not reduced should raise suspicion for disturbed iron utilization rather than absolute iron deficiency. Interpretation of these indices requires particular caution in older patients with multimorbidity and inflammatory states, because ferritin and transferrin may be altered by inflammation (7, 11). In such settings, a panel-based interpretation of iron studies, including TSAT and UIBC in addition to ferritin, together with treatment response, may be more informative than ferritin alone. In the present case, ferritin decreased from 217.8 μg/L at admission to 55.90 μg/L at discharge over approximately 2 weeks, during which the patient’s urinary tract infection and inflammatory state were treated. This trajectory suggests that the initially elevated ferritin level may have been partly influenced by the acute-phase response, thereby providing a practical illustration of why ferritin should be interpreted together with TSAT, UIBC, inflammatory markers, and treatment response rather than in isolation.

Once these diagnostic triggers are recognized, the aim of evaluation should shift from descriptive phenotype-based labeling to mechanism-oriented confirmation. In acquired sideroblastic anemia, bone marrow iron staining with Perls stain and demonstration of ring sideroblasts represent the key morphologic clue, but the presence of ring sideroblasts alone should not be regarded as the end point of diagnosis. Rather, it should be followed by etiologic stratification to distinguish clonal from non-clonal causes. Contemporary WHO and ICC frameworks further emphasize that ring sideroblast-associated phenotypes may overlap with genetically defined entities, particularly those related to SF3B1; therefore, morphologic findings should be interpreted in conjunction with molecular and other diagnostic criteria (12, 13).

The presence of ring sideroblasts should prompt etiologic stratification rather than immediate assignment to a clonal or non-clonal category. Contemporary WHO and ICC frameworks emphasize that interpretation should integrate morphology, cytopenia pattern, blast percentage, cytogenetics, molecular findings, medication and exposure history, nutritional status, and longitudinal follow-up. In the present case, the available data did not justify a definitive diagnosis of MDS, but they also did not prove a non-clonal etiology. Therefore, the case was interpreted as an acquired ring sideroblast phenotype of undetermined etiology requiring longitudinal clonal surveillance.

### 3.2 Comparison with prior pyridoxine-responsive or reversible RS cases

Previous reports have shown that pyridoxine responsiveness may occur in selected patients with ring sideroblast phenotypes. Cotter et al. (14) described two pyridoxine-responsive patients who were initially diagnosed with acquired refractory anemia with ring sideroblasts but were subsequently found to carry ALAS2 mutations, supporting late-onset X-linked sideroblastic anemia rather than a purely acquired clonal disorder. Similar late-onset presentations have also been reported in older patients, including an 81-year-old hemodialysis patient in whom pyridoxine eliminated ring sideroblasts and ALAS2 mutation testing confirmed the diagnosis (15). In addition, reversible acquired causes should also be considered. Nakao et al. (16) recently reported an adult patient with antidepressant-associated vitamin B6 deficiency and ring sideroblasts, whose anemia improved after pyridoxine supplementation and discontinuation of the offending drug. These reports emphasize that ring sideroblasts should be interpreted together with medication exposure, nutritional status, molecular findings, and longitudinal follow-up before assigning the disease to a clonal myeloid category.

Furthermore, Zhang et al. recently reported a 78-year-old male patient with chronic obstructive pulmonary disease who developed severe drug-induced acquired sideroblastic anemia after combined olanzapine and fluvoxamine therapy, with a nadir hemoglobin level of 37 g/L and 19% ring sideroblasts on bone marrow iron staining (17). This report indicates that, in older patients with multiple comorbidities, medication-related non-clonal triggers may give rise to a severe ring sideroblast phenotype. In comparison, the present case is notable for the patient’s advanced age, multiple comorbidities, severe anemia at presentation, and sustained hematologic stability after discontinuation of unnecessary iron exposure and multifactorial supportive management including pyridoxine supplementation.

### 3.3 Limitations and future directions

This report has several limitations. First, the etiology of the ring sideroblasts was not conclusively established. Cytogenetic analysis, expanded myeloid NGS, serum copper/zinc assessment, baseline pyridoxal 5′-phosphate measurement, ALAS2 germline testing, and repeat bone marrow evaluation were not available because of insurance coverage limitations, patient economic burden, and real-world clinical feasibility. Therefore, copper deficiency, zinc-related copper deficiency, late-onset inherited sideroblastic anemia, SF3B1-negative MDS, and clonal hematopoiesis could not be definitively excluded. Second, the marrow description was limited by the retrospective nature of this case. A complete lineage-by-lineage dysplasia assessment and explicit morphologic blast percentage were not available in the original marrow report. Third, hematologic improvement could not be attributed to pyridoxine alone because the patient also received transfusion support, erythropoietin, infection treatment, discontinuation of iron therapy, and optimization of comorbidities. Therefore, this case should be interpreted as a clinically instructive example of iron-refractory microcytic anemia with ring sideroblasts, rather than as definitive mechanistic evidence of pyridoxine-responsive non-clonal sideroblastic anemia. Continued follow-up, repeat marrow evaluation, cytogenetic analysis, and expanded molecular testing should be considered if cytopenias recur, persist, or worsen.

## 4 Conclusion

This case indicates that microcytic hypochromic anemia in older adults should not be empirically assumed to represent iron deficiency anemia. In patients with poor response to iron therapy and iron indices discordant with an iron-deficient phenotype, early diagnostic reassessment is essential. Bone marrow iron staining, identification of ring sideroblasts, and appropriate stratified testing are pivotal in this setting. These evaluations not only help distinguish clonal from non-clonal etiologies and avoid unnecessary iron exposure, but also inform subsequent adjustment of targeted treatment strategies, ultimately improving patient outcomes.

## Data Availability

The original contributions presented in the study are included in the article/Supplementary material, further inquiries can be directed to the corresponding authors.
