# Supplementary material for: A diagnostic pitfall in iron-refractory microcytic hypochromic anemia with acquired ring sideroblasts initially treated as iron deficiency anemia—a case report
Source: Front Med (Lausanne). 2026 Jun 8;13:1838995. doi: 10.3389/fmed.2026.1838995 (PMC13283894; doi:10.3389/fmed.2026.1838995)
Supplement: Supplementary file 1 [file Table_1.docx]

**Supplementary Table S1.** Medication, exposure, nutritional, and family history review relevant to the differential diagnosis of the acquired ring sideroblast phenotype.

| **Category** | **Medication/exposure reviewed** | **Dose and frequency** | **Timing/status** |
| --- | --- | --- | --- |
| Type 2 diabetes mellitus | Insulin aspart injection | 14 units subcutaneously before each meal | Long-term treatment before admission |
|  | Insulin glargine injection | 30 units subcutaneously once daily at 22:00 | Long-term treatment before admission |
|  | Linagliptin tablets | 5 mg orally once daily in the morning | Long-term treatment before admission |
| Hypertension | Amlodipine besylate tablets | 5 mg orally once daily in the morning | Long-term but irregular treatment before admission |
| Urinary tract infection / infection management | Levofloxacin sodium chloride injection | 0.5 g by intravenous infusion once daily | During hospitalization |
| Prior empiric anemia treatment | Polysaccharide-iron complex | 0.1 g orally once daily | Approximately 1 month before admission |
| Antitubercular drugs | Isoniazid | Not used | No exposure identified |
| Antibiotics associated with sideroblastic anemia | Linezolid; chloramphenicol | Not used | No exposure identified |
| Psychotropic medications | Antidepressants or other psychotropic drugs previously associated with sideroblastic anemia | Not used | No exposure identified |
| Toxic exposure | Alcohol, lead, or other known toxins | Denied / not identified | Before admission |
| Zinc-related exposure / copper deficiency risk | Zinc-containing supplements; denture adhesives | Not used / not identified | Before admission |
| Nutritional deficiency | Vitamin B12 and folate status | Vitamin B12: 259.977 pg/mL, reference range 200–900 pg/mL; folate: 12.438 nmol/L, reference range 6.8–36.3 nmol/L | At admission |
| Hereditary anemia history | Family history of hereditary anemia or hematologic disease | No relevant family history reported | History taking |
