# Supplementary material for: A diagnostic pitfall in iron-refractory microcytic hypochromic anemia with acquired ring sideroblasts initially treated as iron deficiency anemia—a case report
Source: Front Med (Lausanne). 2026 Jun 8;13:1838995. doi: 10.3389/fmed.2026.1838995 (PMC13283894; doi:10.3389/fmed.2026.1838995)
Supplement: Supplementary file 2 [file Table_2.docx]

**Supplementary Table S2**. Etiologic stratification of the ring sideroblast phenotype in the present case.

| **Etiologic category** | **Evaluation considered** | **Findings in this case** |
| --- | --- | --- |
| Typical iron deficiency anemia | Iron profile and response to iron therapy | Serum iron 37.6 μmol/L, TSAT 79.8%, UIBC 9.5 μmol/L, ferritin 217.8 μg/L, no improvement after oral iron |
| Inflammation-related ferritin distortion | ESR, hs-CRP, infection status, ferritin trend | ESR 119 mm/h, hs-CRP 9.98 mg/L, urinary tract infection treated, ferritin decreased to 55.90 μg/L at discharge |
| Drug-associated acquired sideroblastic anemia | Medication review | No isoniazid, linezolid, chloramphenicol, antidepressants, or psychotropic drugs identified |
| Alcohol or toxic exposure | Exposure history | Alcohol, lead, and other toxic exposures denied or not identified |
| Zinc-related copper deficiency | Zinc supplement and denture adhesive history, copper/zinc testing | No zinc supplements or denture adhesive exposure reported; copper/zinc levels not measured |
| Vitamin B12 or folate deficiency | Vitamin B12 and folate levels | Vitamin B12 and folate within laboratory reference ranges |
| Vitamin B6 deficiency | Baseline PLP level | Not measured |
| Late-onset inherited sideroblastic anemia | Family history and ALAS2 germline testing | No family history of hereditary anemia; ALAS2 germline testing not performed |
| Clonal myeloid disease / MDS | Morphology, blast assessment, SF3B1, cytogenetics, expanded myeloid NGS | SF3B1 negative; CD34-positive cells 0.25% by flow cytometry; cytogenetics and expanded NGS not available |
| Longitudinal clinical behavior | Blood count follow-up and repeat marrow if indicated | Hb stable at approximately 3 and 6 months without further transfusion |
