# Supplementary material for: A diagnostic pitfall in iron-refractory microcytic hypochromic anemia with acquired ring sideroblasts initially treated as iron deficiency anemia—a case report
Source: Front Med (Lausanne). 2026 Jun 8;13:1838995. doi: 10.3389/fmed.2026.1838995 (PMC13283894; doi:10.3389/fmed.2026.1838995)
Supplement: Supplementary file 3 [file Table_3.docx]

**Supplementary Table S3.** Treatment timeline and interpretation of hematologic changes.

| Phase | Main interventions | Hemoglobin change | Interpretation |
| --- | --- | --- | --- |
| Admission | Iron discontinued; diagnostic reassessment initiated | Hb 46 g/L | Severe symptomatic anemia with iron indices inconsistent with typical IDA |
| Hospital days 2–3 | 5 units leukoreduced packed red blood cells | Hb subsequently increased to 93 g/L by hospital day 7 | Early Hb increase largely compatible with transfusion-related increment |
| Hospitalization | Erythropoietin, intravenous pyridoxine, infection treatment, glycemic optimization, electrolyte correction, antihypertensive therapy | Hb 104 g/L at discharge | Multifactorial treatment phase; individual treatment effects cannot be separated |
| Post-discharge | Oral pyridoxine maintenance and comorbidity management; no further transfusion | Hb 112 g/L at approximately 3 months | Transfusion-free hematologic stability |
| Follow-up | Continued outpatient management; no further transfusion | Hb 118 g/L at approximately 6 months | Sustained hematologic stability, but not definitive proof of pyridoxine-specific efficacy |
