# Supplementary material for: A diagnostic pitfall in iron-refractory microcytic hypochromic anemia with acquired ring sideroblasts initially treated as iron deficiency anemia—a case report
Source: Front Med (Lausanne). 2026 Jun 8;13:1838995. doi: 10.3389/fmed.2026.1838995 (PMC13283894; doi:10.3389/fmed.2026.1838995)
Supplement: Supplementary file 4 [file Table_4.docx]

**Supplementary Table S4** Additional negative screening results not included in the main table.

| **Category** | **Parameter** | **Result** | **Unit** | **Reference range / interpretation** |
| --- | --- | --- | --- | --- |
| Metabolic status | Random plasma glucose | 9.96 | mmol/L | 3.9–6.1 |
|  | Urine ketones | Positive | — | Negative |
|  | Hemoglobin A1c | 9.2 | % | 4.0–6.0 |
| Electrolytes | Sodium | 137.3 | mmol/L | 135–145 |
|  | Potassium | 3.48 | mmol/L | 3.5–5.3 |
|  | Calcium | 2.23 | mmol/L | 2.0-2.7 |
| Autoimmune screening | Antinuclear antibody | 1.92 | RU/mL | 0–20 |
|  | ANA-associated autoantibody panel | Within reference range | — | Within reference range |
|  | Antineutrophil cytoplasmic antibody panel | Negative | — | Negative |
| Endocrine screening | Thyroid function and thyroid autoantibodies | Within reference range | — | Within reference range |
| Other | Creatine kinase | 17 | U/L | 48-165 |
