# Supplementary material for: A diagnostic pitfall in iron-refractory microcytic hypochromic anemia with acquired ring sideroblasts initially treated as iron deficiency anemia—a case report
Source: Front Med (Lausanne). 2026 Jun 8;13:1838995. doi: 10.3389/fmed.2026.1838995 (PMC13283894; doi:10.3389/fmed.2026.1838995)
Supplement: Supplementary file 5 [file Table_5.docx]

**Supplementary Table S5.** Bone marrow smear findings.

| Category | Item | Finding |
| --- | --- | --- |
| Specimen and cellularity | Specimen quality | Bone marrow sampling, smear preparation, and staining were good; marrow particles (+) and fat droplets (+) were observed. |
| Specimen and cellularity | Bone marrow cellularity | Markedly active marrow |
| Differential count | Counted nucleated marrow cells | 200 nucleated marrow cells |
| Differential count | G:E ratio | 0.75:1, indicating erythroid-predominant hyperplasia |
| Differential count | Morphologic blast assessment | No blasts were recorded in the 200-cell bone marrow differential count; no increase in blasts was reported. |
| Granulocytic lineage | Overall finding | Markedly active granulopoiesis; granulocytic cells at different maturation stages showed no obvious morphologic abnormalities. |
| Granulocytic lineage | Approximate proportion | Neutrophilic granulocytic cells accounted for approximately 39.5% of nucleated marrow cells, with an additional 0.5% eosinophilic metamyelocytes recorded. |
| Erythroid lineage | Overall finding | Markedly active erythropoiesis, mainly composed of intermediate and late erythroblasts. |
| Erythroid lineage | Approximate proportion | Approximately 53.0% of nucleated marrow cells. |
| Erythroid lineage | Morphology | A small number of erythroblasts with nuclear irregularities were observed; mature erythrocytes showed anisocytosis with occasional polychromatophilic red cells. |
| Megakaryocytic lineage | Megakaryocytes | A total of 58 megakaryocytes were counted, including immature megakaryocytes 6%, granular megakaryocytes 78%, platelet-producing megakaryocytes 14%, and naked nuclei 2%; platelet clusters were readily observed. |
| Megakaryocytic lineage | Morphologic abnormality | No obvious megakaryocytic morphologic abnormality was reported. |
| Iron staining | Extracellular iron | Markedly increased (+++) |
| Iron staining | Intracellular iron | Type I 26%, type II 15%, type III 13%, and type IV 5%. |
| Iron staining | Ring sideroblasts | 14% of erythroid precursors. |
| Other cells | Lymphoid cells | Lymphocytes accounted for approximately 4%, including 0.5% immature lymphocytes. |
| Other cells | Monocytes | 3%. |
| Other cells | Plasma cells / abnormal cells | No abnormal lymphoid cells, plasma cell expansion, myeloma cells, or other abnormal cells were recorded in the differential table. |
| Overall impression | Morphologic summary | Markedly active marrow with erythroid predominance and 14% ring sideroblasts. A small number of erythroblasts with nuclear irregularities were observed, while no obvious abnormalities were reported in the remaining cell lineages. Correlation with clinical findings, immunophenotyping, and genetic testing was recommended. |
